# Comparing Safety and Efficacy Outcomes of Gastric Bypass and Sleeve Gastrectomy in Patients With Type 2 Diabetes Mellitus: A Systematic Review and Meta-Analysis

**DOI:** 10.7759/cureus.52796

**Published:** 2024-01-23

**Authors:** Mohamed Elsaigh, Bakhtawar Awan, Ahmed Shabana, Azka Sohail, Ahmad Asqalan, Omnia Saleh, Justyna Szul, Rana Khalil, Hatem Elgohary, Mohamed Marzouk, Mohamed Alasmar

**Affiliations:** 1 General and Emergency Surgery, Northwick Park Hospital, London North West University, London, GBR; 2 Bariatric and General Surgery, Shifa Hospital, Cairo, EGY; 3 Thoracic Surgery, Norfolk and Norwich University Hospital, Norwich, GBR; 4 Surgery, Brigham and Women's Hospital, Boston, USA; 5 General and Emergency Surgery, Newcastle University Hospitals and Kasralainy Medical School, Cairo University, Cairo, EGY; 6 General and Emergency Surgery, Helwan University, Cairo, EGY; 7 General and Emergency Surgery, Salford Royal Hospital, University of Manchester, Manchester, GBR

**Keywords:** weight loss and obesity, systematic review and meta analysis, gastric sleeve surgery, gastric bypass surgery, type2 diabetes mellitus

## Abstract

Sleeve Gastrectomy (SG) could be done by the removal of a big portion of the stomach, leading to reduced amounts of food taken as a result of the smaller stomach size. In contrast, Roux-en-Y Gastric Bypass (RYGB) can be done by creating a small stomach pouch and rerouting a part of the small intestine, employing combined mechanisms of restriction and malabsorption to limit food intake and modify nutrient absorption. Our aim is to identify the most effective and safest surgical intervention for individuals with both Type 2 Diabetes Mellitus (T2DM) and obesity, considering both short and long-term outcomes. We will assess participants undergoing either SG or RYGB to determine the optimal surgical approach. We made a thorough search of PubMed, Cochrane Library, Scopus, and Web of Science databases up to November 2023. Our focus was on randomized controlled trials (RCTs) comparing the safety and efficacy of RYGB and SG in T2DM regarding any extractable data. We excluded studies of other designs, such as cohorts, case reports, case series, reviews, in vitro studies, postmortem analyses, and conference abstracts. Utilizing Review Manager 5.4, we performed a meta-analysis, combining risk ratios (RR) with a 95% confidence interval (CI) conducted for binary outcomes, while mean with SD and 95% CI are pooled for the continuous ones. The total number of participants in our study is 4,148 patients. Our analysis indicates superior outcomes in the group undergoing RYGB surgery compared to the SG group (RR = 0.76, 95% (CI) (0.66 to 0.88), P = 0.0002). The pooled data exhibited homogeneity (P = 0.51, I2 = 0%) after employing the leave-one-out method. For the 1-3 year period, six studies involving 332 patients with T2DM yielded non-significant results (RR = 0.83, 95% CI (0.66 to 1.06), P = 0.14) with homogeneity (P = 0.24, I2 = 28%). Conversely, the 5-10 year period, with six studies comprising 728 DM patients, demonstrated significant results (RR = 0.69, 95% CI (0.56 to 0.85), P = 0.14) and homogeneity (P = 0.84, I2 = 0%). In terms of total body weight loss, our findings indicate significantly higher weight loss with RYGB (mean difference (MD) = -6.13, 95% CI (-8.65 to -3.6), P > 0.00001). However, pooled data exhibited considerable heterogeneity (P > 0.00001, I2 = 93%). Subgroup analyses for the 1-3 year period (five studies, 364 DM patients) and 5-10 year period (six studies, 985 DM patients) also revealed significant differences favoring RYGB, with heterogeneity observed in both periods (1-3 years: P > 0.00001, I2 = 95%; 5-10 years: P = 0.001, I2 = 75%). RYGB demonstrated significant long-term improvement in diabetes remission and superior total body weight loss compared to SG. While no notable differences were observed in other efficacy outcomes, safety parameters require further investigation. no significant distinctions were found in any of the safety outcomes: hypertension (HTN), high-density lipoprotein (HDL), hyperlipidemia, fasting blood glucose, vomiting, low-density lipoprotein (LDL), and total cholesterol. Further research is essential to comprehensively assess safety outcomes for both surgical approaches.

## Introduction and background

The worldwide frequency of obesity and diabetes mellitus (DM) is consistently growing [1]. More than 39% of adults are overweight according to WHO in 2016 and 13% are obese [2]. Each increase in BMI above the usual range is associated with increased mortality, with a BMI above 40 reducing life expectancy by eight to ten years [3]. There is an association between Type 2 DM (T2DM) and obesity; As obesity significantly impacts the early development of chronic diseases, including T2DM, cancer, and cardiovascular diseases. Additionally, it can cause functional impairments, frailty, and an increase in hospitalizations [4-6]. Additionally, obesity increases insulin resistance and affects the body's ability to keep blood sugar levels regulated, contributing to the onset of the disease [7].

Both obesity and T2DM have treatments available, but most of them have the opposite effect on each other. Nonetheless, when it comes to treating both conditions effectively, surgical procedures, especially bariatric surgery, are the way to go [8,9]. New guidelines are recommended for the safe and effective use of bariatric surgery in a wide variety of populations, including children and adolescents, based on specific criteria [10].

As per the American Society for Metabolic and Bariatric Surgery, Sleeve Gastrectomy (SG) emerged as the predominant bariatric surgical procedure in the U.S. in 2017, representing 59.4% of all such surgeries. Following closely, the second most prevalent method was Roux-en-Y Gastric Bypass (RYGB), comprising 17% of the total bariatric surgeries performed [11]. The top two most commonly used bariatric techniques worldwide are considered RYGB and SG [12]. In 2016, SG surgery was performed more often than any other type of surgery worldwide [13].

Both procedures made considerable weight loss and remission of obesity-related comorbidities [14]. The specific mechanism is not entirely understood, but it is believed that weight loss and a decrease in appetite are achieved by decreasing the size of the stomach. Additionally, the improvement in bile acids is more pronounced in this procedure. Nonetheless, it is crucial to note that only RYGB incorporates duodenal bypass, a distinctive feature believed to augment diabetes remission by triggering the production of incretin hormones [15]. The modification of the gastrointestinal anatomy in RYGB, specifically involving the duodenum, is thought to play a role in the observed improvements in diabetes outcomes, possibly through enhanced incretin hormone activity [16-19]. Some researchers suggest that improved insulin sensitivity in the liver, especially in the weeks following surgery, might contribute to the prompt improvement of glucose intolerance [20]. While RYGB is acknowledged as the premier metabolic surgery, it is linked with potential complications, including but not limited to marginal ulcers, internal hernias, dumping syndrome, malnutrition, and deficiencies in essential vitamins [21,22].

In reviews, SG had fewer postoperative complications and reoperations, but more reflux symptoms, such as gastroesophageal reflux disease (GERD) and esophagitis, compared to other procedures [23,24]. On the other hand, RYGB was superior in secondary outcomes like dyslipidemia and hypertension (HTN) [25,26]. The comparison of the two operations regarding their efficacy in improving DM and facilitating weight loss has not yielded any established differences. The current study, therefore, aims to primarily evaluate the mentioned variables for a follow-up duration of up to 10 years. By conducting this study, we aim to identify the most effective and safest surgical intervention for patients who were selected for SG or RYGB with T2DM and obesity in the short and long terms.

## Review

Methods

The researchers conducted a comprehensive and thorough evaluation of a systematic review and meta-analysis, ensuring that they followed the most up-to-date guidelines and standards set by PRISMA and Cochrane [27,28]. 

Literature Search and Keywords

We searched PubMed, Cochrane Library, Scopus, and Web of Science databases for relevant trials until November 2023 Our search strategy (("type 2 diabetes" OR "Non-insulin-dependent diabetes mellitus" OR "Adult-onset diabetes" OR "Late-onset diabetes" OR "Insulin-resistant diabetes" AND "Roux-en-Y gastric bypass" OR "RYGB" OR "Roux-en-Y" OR "RYGB Surgery" OR "RYGBP" OR "Roux-en-Y RNY Surgery" OR "RYGB Weight Loss Surgery" AND "VSG" OR "sleeve gastrectomy" OR "vertical sleeve gastrectomy" OR "LSG")).

Eligibility Criteria and Study Selection

We compared RYGB and SG in randomized controlled trials (RCTs) for safety and efficacy outcomes in T2DM patients. We eliminated duplicates with EndNote, screened titles and abstracts, and conducted full-text screening for eligible studies. We also searched references for relevant studies. The previous steps were done by two authors, and any conflicts were decided by the last author. We excluded the following designs; cohort, case reports, case series, reviews, editorials, in vitro, postmortem, and conference abstracts. We excluded any other indication of the operations other than DM patients.

Quality and Risk of Bias (ROB)

To evaluate the degree of bias in the studies under consideration, the team used the Cochrane Handbook for Systematic Reviews (version 1). The studies were categorized as having a high, low, or unclear ROB. Any uncertainties were resolved through conversation and consensus with two co-authors.

Data Extraction

From the included trials, two authors extracted general baseline and summary data that included country, study design, study arms, age, sex, weight, BMI, HbA1c, follow-up duration, inclusion criteria, and study conclusions into an Excel sheet. Outcomes were divided into efficacy and safety sections. In efficacy outcomes, we focused on diabetes remission, HbA1c, total body weight loss, BMI, and quality of life (QOL). On the other hand safety outcomes were fasting glucose level, dyslipidemia, total cholesterol, high-density lipoprotein (HDL), low-density lipoprotein (LDL), HTN, and vomiting.

Analysis

We employed Review Manager 5.4 software for data analysis, using risk ratio (RR) or mean difference (MD) with 95% confidence intervals (CI) based on the nature of the data (dichotomous or continuous). Statistical significance was considered at a P-value below 0.05. Heterogeneity was assessed using I-square (I2) and chi-square tests. For homogeneous data (P ≥ 0.1 or I2 < 50%), a fixed-effect model was used, while for heterogeneous data (P < 0.1 or I2 > 50%), a random-effect model was applied. Subgroup analyses were conducted based on follow-up duration.

Results

Literature Search and Study Selection 

**Figure 1:**
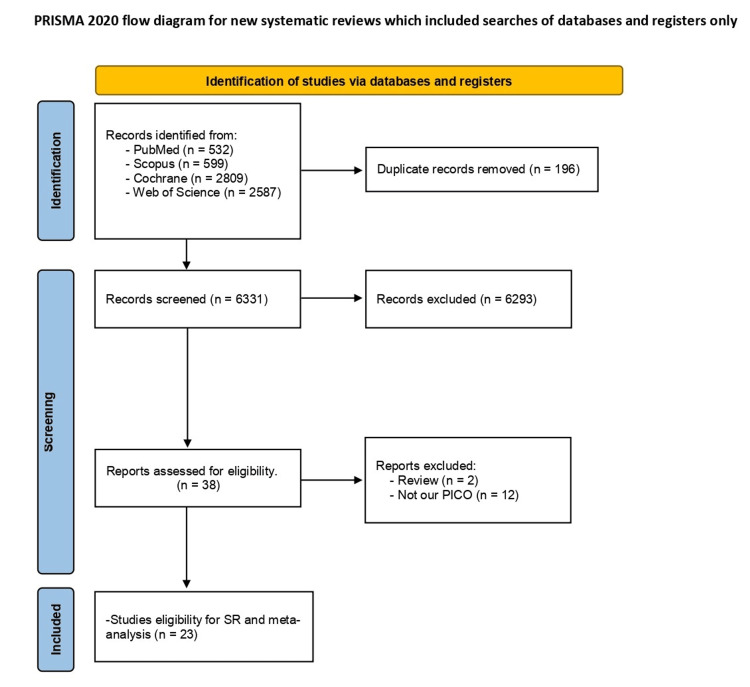
PRISMA Flow diagram

After conducting an extensive literature search on various search engines, we retrieved 6,331 records. We excluded 196 papers by removing duplicates and further excluded 6293 articles based on title and abstract screening. Out of the remaining 38 articles, we conducted full-text screening and shortlisted 23 RCTs that matched our standards. We included these 23 RCTs in the meta-analysis to obtain evidence, out of which seven were only in narrative form [23-51]. All the data are shown in Figure 1.

Study Characteristics

We reviewed all RCTs that investigated the effectiveness of two types of weight loss surgeries, SG and RYGB, on 4,148 confirmed T2DM patients across various countries including the USA, Europe, Taiwan, Brazil and New Zealand. The sample size of both the groups varied from 15 to 462 participants. The female population was more prominent than males. The researchers specified the follow-up duration either by a period ranging from one year to ten years on most of the outcomes. The baseline and summary characteristics of all the included RCTs are presented in Table 1.

**Table 1:** Baseline and summary [29-51] RCT: Randomized controlled trial; T2D: Type 2 diabetes; T2DM: Type 2 diabetes mellitus; SG: Sleeve gastrectomy; RYBG: Roux-en-Y gastric bypass; HbA1c: Glycated hemoglobin; QoL: Quality of life; HDL: High-density lipoprotein; LDL: Low-density lipoprotein; LSG: Laparoscopic sleeve gastrectomy, SR: Systematic review

Study ID	Intervention	Country	Study design	Study arms, N	Male, N (%)	Age, Mean (SD)	BMI, Mean (SD)	HbA1c%	Bodyweight, kg	Follow-up duration (Year)	Primary outcome	Inclusion criteria	Conclusion
[29] Sleeve 2023	SG	UK	RCT	420	99(23.4)	47.8 ±11.0	46.1±6.9	NA	128.8±23.8	NA	1-Fasting glucose, 2-Hypertension, 3-Total cholesterol, 4-QoL	1-Patients with ages ranging from 18 and 60 years, 2-BMI 35–43 kg/m^2^, 3-T2D on hypoglycemic agents alone, insulin, or both	“By-Band-Sleeve fully recruited. Participant characteristics are consistent with contemporary patients having bariatric surgery, and therefore the results will be generalizable”
	gastric bypass			462	117(23.2)	47.4 ±10.3	46.9±7.1	NA	131.4 ±23.8				
Capristo 2018 [30]	SG	Italy	RCT	60	NA	NA		38.17 ± 3.13		1	1-Fasting glucose, 2-LDL, 3-Total cholesterol	1-Age from 25 to 65 years, 2-BMI of 40 or 35 to 40 kg/m^2^ in the presence of obesity complications, 3-The ability to understand and comply with the study process	"We demonstrate that reactive hypoglycemia is equally prevalent following sleeve gastrectomy (SG) and does not present a safer alternative compared to Roux-en-Y gastric bypass (RYGB). However, RYGB is linked to more pronounced episodes of hypoglycemia."”
	gastric bypass			60	NA	NA		40.0 ±7.40					
Casajoana 2017 [31]	SG	Spain	RCT	15	5 (33.3)	49.20 ± 9.16		7.89 ± 1.71		1	1-HDL, 2-HbA1c, 3-Total body loss	1-Patients with ages ranging from 18 and 60 years, 2-BMI 35–43 kg/m^2^, 3-T2D on hypoglycemic agents alone, insulin, or both	"At the 12-month mark, MRYGB demonstrates superior weight loss and Type 2 Diabetes (T2D) remission rates, attributed to increased GLP-1 secretion."
	gastric bypass			15	7 (46.6)	51.10 ± 7.70		7.39 ± 1.95					
Fatima 2022 [32]	SG	Norway	RCT	53	NA	48 ±10		NA		1	1-Fasting glucose, 2-Diabetes remission, 3-HbA1c, 4-Total body loss	1-≥ 18 years, 2-Current BMI ≥ 33.0 kg/m^2^ with previously verified BMI ≥ 35.0 kg/m^2^, and T2D, 3-HbA1c ≥ 48 mmol/mol (6.5%) or use of antidiabetic medications with HbA1c ≥ 43 mmol/mol (6.1%)	“RYGB was associated with greater improvement in β-cell function and higher postprandial GLP-1 levels than SG”
	gastric bypass			53	NA			NA					
Hofsø 2019 [33]	SG	Norway	RCT	55	23 (42)	47·1 ±10·2	42·1±5·3	7.2±2.2	126·7 ±21·4	1	1- Fasting glucose,2-HDL,3- LDL,4- Total cholesterol, 5- BMI,6- Diabetes remission,7- HbA1c	1-18 years or older, 2 current BMI of 33·0 kg/m² or higher with previously verified BMI of 35·0 kg/m² or higher, 3-T2D (HbA1c of ≥6·5% (48 mmol/mol) or use of (43 mmol/mol)). Antidiabetic medications with HbA1c of ≥6·1%	“"At the one-year post-surgery mark, gastric bypass demonstrated superiority over sleeve gastrectomy in achieving type 2 diabetes remission, while both procedures exhibited a comparable positive impact on β-cell function. Opting for gastric bypass as the primary bariatric procedure for individuals with obesity and type 2 diabetes has the potential to enhance diabetes management and lower associated societal expenses."
	gastric bypass			54	14 (26)	48·2 ± 8·9	42·4 ±5·4	7·6± 1.2	124·4 ±23·2				
Kalinowski 2017 [34]	SG	Poland	RCT	36	26 (27.8)	44.9 ±10.6	46.1 ±5.9	6.4±1.3	127.9±17.6	1	1- Dyslipidimia, 2-Fasting glucose, 3-Hypertension, 4-BMI, 5- Diabetes remission, 6- HbA1c, 7- Total body loss	1-BMI ≥40 kg/m^2^ or BMI ≥35 kg/m^2^ with at least one comorbidity associated with obesity, 2-Age range 18-60 years	"RYGB and SG result in similar weight loss and enhanced glucose metabolism. While ghrelin levels decrease after SG and increase after RYGB, this variation does not impact the comparable outcomes of these procedures during a one-year follow-up. The role of ghrelin in influencing weight loss or metabolic improvements post-bariatric surgery is complex, influenced by various factors."
	gastric bypass			36	23 (26.1)	43.9 ±10.8	48.6 ±5.4	6.3±0.9	141.2±20.6				
Laurenius 2023 [35]	SG	Sweden	RCT	31	NA	47 ± 11	40.5±4.1	NA	118.9 ±19.6	5	1-BMI, 2- Total body loss	1-BMI of 35 to 50 kg/m^2^, 2-Age of 18 to 60 years, 3-T2D requiring any glucose-lowering pharmacologic medication, not dietary treatment alone	"After five years post-surgery, individuals assigned to Roux-en-Y gastric bypass (RYGB) reported significantly increased food intake in comparison to those who underwent sleeve gastrectomy (SG), despite having lower body weight. Further investigation is required to understand the reasons behind and the significance of the elevated food intake following RYGB compared to SG."
	gastric bypass			29	NA	49±9	39.8±3.9	NA	119.3 ±15.2				
Murphy 2022 [36]	SG	New Zealand	RCT	58	NA	45.5 ±6.4	32 ±55	7.8 ±1.2	126. ±24.5	5	1-Vomiting, 2-BMI, 3-Diabetes remission	1-Age between 20–55 years, 2-T2D of six months duration or more, 3-BMI of 35–65 kg/m^2^ for 5 years minimally	“SR-LRYGB provided superior diabetes remission and weight loss compared with LSG at 5 years, with similar low risks of complications”
	gastric bypass			56	NA	46.6 ±6.7	23 ±41	8.1 ±1.7	123.4±21.3				
Pajecki 2023[37]	SG	Brazil	RCT	18	0	68 ± 2.8	43.1±1.2	NA	109.9±4.1	3	1-HDL, 2-LDL, 3-BMI, 4-HbA1c, 5-Total body loss	Obesity in patients>65 years	“LYRGB is associated with more significant weight loss and improvement of glycated hemoglobin and lipid levels after 3 years. Recent literature has shown similar weight loss between procedures on short-term follow-up in this population”
	gastric bypass			18	5 (27.8)	67 ± 1.9	46.89±1.1	NA	118.0±4.2				
Peterli 2018 [38]	SG	Switzerland	RCT	107	30 (28)	43.0 ±11.1	43.6 ±5.2	NA	123.5 ±19.4	5	1-Dyslipidimia, 2-HDL, 3-Hypertension, 4-LDL, 5- Diabetes remission, 6-QoL, 7-Total body loss	1-BMI greater than 40 years or greater than 35 with the presence of at least one comorbidity, 2-An age of 18 up to 65 years, 3-Failure of treatment for two years	"Among individuals with morbid obesity, there was no statistically significant distinction in excess BMI loss between laparoscopic sleeve gastrectomy and laparoscopic Roux-en-Y gastric bypass at the five-year post-surgery follow-up."
	gastric bypass			110	31 (28.2)	42.1 ±11.2	44.2±5.3	NA	124.8±19.8				
Pullman 2023 [39]	SG	New Zealand	RCT	39	23 (59)	46.7 ±6.6	41.7 ±6.1	7.7 ±1.1	117.6 ±20.6	7	1-Vomiting, 2-BMI, 3-Diabetes remission	1-20–55 years, 2-BMI of 35–65 kg/ m^2^, 3-T2D (as defined by the American Diabetes Association) for at least 6 months duration, 4-Suitable for either procedure and committed to follow-up	“SR-LRYGB was superior to LSG for diabetes remission and weight loss at 7 years following surgery, with acceptable complication rates”
	gastric bypass			50	21 (42)	48.4±5.4	43.6 ±7.2	8.1 ±1.7	125.4 ±22.6				
Salminen 2022 [40]	SG	Finland	RCT	121	34 (28.1)	48.5 ±9.6	48.5±9.6	NA	130.1±21.5	10	1-Dyslipidimia, 2-Hypertension, 3-Vomiting, 4-BMI, 5-Diabetes remission	1-An age ranging from 18 to 60 years, 2-BMI 40 and higher or 35 and higher with significant comorbidity related to obesity, 3-Previously failed treatments	“At 10 years, %EWL was greater after LRYGB and the procedures were not equivalent for weight loss, but both LSG and LRYGB resulted in good and sustainable weight loss. Esophagitis was more prevalent after LSG, but the cumulative incidence of BE was markedly lower than in previous trials and similar after both procedures”
	gastric bypass			119	39 (32.8)	48.4±9.3	48.4±9.3	NA	134.9 ±22.5				
Svanevik 2023[41]	SG	Norway	RCT	55	23 (42)	47∙1±10∙2	42∙1 ±5∙3	7.9±5.5	126∙7±21∙4	3	1-BMI, 2-Diabetes remission, 3-HbA1c, 4-QoL, 5-Total body loss	1-Age over 18 years. 2- patients with type 2 diabetes 3- current BMI 33·0 kg/m² or greater, 4- Diabetes was diagnosed if glycated hemoglobin was at least 6·5% (48 mmol/mol)	“"After three years, gastric bypass proved to be more effective than sleeve gastrectomy for individuals with type 2 diabetes and obesity concerning aspects such as weight-related quality of life, reflux symptoms, weight loss, and diabetes remission. However, there were no significant differences between the groups in symptoms such as abdominal pain, indigestion, diarrhea, dumping, depression, and binge eating. This valuable patient-reported information can be utilized in shared decision-making, providing insights into the similarities and distinctions in expected outcomes following the two surgical procedures."
	gastric bypass			54	14 (26)	48∙2±8∙9	42∙4 ±5∙4	7.6±3.1	124∙4±23∙2				
Wallenius 2020 [42]	SG	Sweden	RCT	24	13 (54)	47.0±10.7	40.7±4.2	NA	120±19.2	2	1-Dyslipidimia, 2-Fasting glucose, 3-HDL, 4-Hypertension, 5-LDL, 6-BMI, 7-Diabetes remission, 8-HbA1c, 9-Total body loss	1- T2D requires antidiabetic medications, 2-BMI between 35 and 50 kg/m^2^, 3-Age between 18 and 60 years	“Despite superior excess weight loss after RYGB, T2D remission rates did not differ significantly between RYGB and SG after 2 years. Long-term follow-up data are needed to define the role of SG in the treatment of patients with obesity and T2D”
	gastric bypass			25	13 (52)	49.1 ±9.2	39.3 ±3.6	NA	119 ±15.4				
Wolnerhanssen 2021 [43]	SG	Switzerland	RCT	228	64 (28)	45.9±10.7	45.6±6.5	NA	129.9±22.6	5	1-HDL, 2-LDL, 3-Diabetes remission, 4-QoL, 5-Total body loss	1-BMI greater than 40 or greater than 35 with the presence of at least 1 comorbidity, 2-An age of 18 to 65 years, 3-Failure of treatment for two years	"Although LRYGB induced greater weight loss and better amelioration of hypertension than LSG, there was no difference in remission of T2DM, obstructive sleep apnoea, or QoL at 5 years. There were more complications after LRYGB, but the individual burden for patients with complications was similar after both operations.”
	gastric bypass			229	70 (30.6)	45.3±10.7	46.4±6.6	NA	133.2±24.5				
Yang 2015 [44]	SG	China	RCT	32	9 (28)	40.4 ± 9.4	31.8 ± 3.0	8.5 ± 1.2	88.4 ±6.8	3	1- Fasting glucose,2- HDL,3- LDL,4- Total cholesterol,5- BMI,6- Diabetes remission,7-HbA1c, 8- Total body loss	1-T2DM is established when following a six-month course of medication treatment, HbA1c level remains at or exceeds 7.0%, 2-Individuals meeting the criteria exhibit a BMI falling within the range of 28 to 35 kg/m^2^, 3-Age between 25 and 60 years, 4-A diabetes duration of fewer than ten years	"Over the course of this three-year investigation, sleeve gastrectomy (SG) demonstrated comparable beneficial impacts on diabetes and dyslipidemia when compared to Roux-en-Y gastric bypass (RYGB) in Chinese patients with type 2 diabetes and a BMI falling within the range of 28-35 kg/m2. To validate these findings, further research with longer-term follow-ups and larger sample sizes is essential."
	gastric bypass			32	13 (40)	41.4 ± 9.3	32.3 ± 2.4	8.9 ± 1.3	94.3 ±13.3				
Barstad 2022 [45]	SG	Norway	RCT	55	NA	NA	NA	NA	NA	1	The main aim is the confirmation of gastric bypass superiority in comparison to sleeve gastrectomy.	1-Patients who were 18 years or more, 2-Patients with BMI of 33.0 kg/m^2^ with previously verified BMI of 35 kg/m^2^, 3- atients with T2DM.	"The alterations in dietary fiber and protein intake over the course of one year following both surgical procedures, especially after sleeve gastrectomy (SG), were not aligned with existing dietary recommendations. In practical terms, our results indicate that healthcare professionals and individuals undergoing these surgeries should prioritize achieving adequate levels of protein, fiber, and consider vitamin and mineral supplementation, emphasizing the importance of these nutritional elements."
	gastric bypass			54	NA	NA	NA	NA	NA				
Lee 2011 [46]	SG	Taiwan	RCT	30	NA	45 ±6	NA	NA	NA	1	1-Diabetes remission	1-Patients ranging from 30 to 60 years, 2-BMI of 25 to 35, 3-Poorly controlled HbA1c 7.5%,.4-T2DM and were treated by an endocrinologist for 6 months or longer.	“Participants randomized to gastric bypass were more likely to achieve remission of T2DM. Duo to exclusion plays a role in T2DM treatment and should be assessed”
	gastric bypass			30	NA		NA	NA	NA				
Murphy 2017 [47]	SG	New Zealand	RCT	58	32 (55)	45.5 ± 6.4	41.9 ± 5.9	NA	126.7 ± 24.5	1	1-The proportion of patients achieving HbA1c of less than 42 mmol/mol.	1-Aged 20–55 years, 2-T2D of at least 6 months duration, 3- BMI 35–65 kg/m^2^	“Despite significantly greater weight loss after SRLRYGB, there was similar T2D remission and psychosocial improvement after LSG and SR-LRYGB at 1 year”
	gastric bypass			56	23 (41)	46.6 ± 6.7	42.2 ± 6.2	NA	123.4 ± 21.3				
Peterli 2017 [48]	SG	Switzerland	RCT	107	30 (28)	43.0 ± 11.1	43.6 ± 5.3	NA	123.5 ±19.4	1	1- Fasting glucose	1-BMI >40 or >35 kg/m2 with the presence of at least one comorbidity, 2-Age between 18 and 65 years, 3-Failure of conservative treatment over 2 years	“LSG was associated with shorter operation time and a trend toward fewer complications than with LRYGB. Both procedures were almost equally efficient regarding weight loss, improvement of comorbidities, and quality of life 1 year after surgery. Long-term follow-up data are needed to confirm these facts.”
	gastric bypass			118	31 (28)	42.1 ± 11.2	44.2 ± 5.3	NA	124.8±19.8				
Salminen 2018 [49]	SG	Finland	RCT	120	34 (28.1)	48.5 ±9.6	45.5 ±6.2	NA	130.1±21.5	5	1-The excess weight loss from initial estimation.	1- Age of 18 to 60 years, 2-BMI greater than 40 or greater than 35 with significant obesity-associated comorbidity	"Among individuals with morbid obesity, the application of laparoscopic sleeve gastrectomy, as opposed to laparoscopic Roux-en-Y gastric bypass, did not satisfy the criteria for equivalence in percentage excess weight loss after 5 years. While gastric bypass exhibited a higher percentage of excess weight loss compared to sleeve gastrectomy at the 5-year mark, the observed difference did not reach statistical significance, as per the predetermined equivalence margins."
	gastric bypass			119	39 (32.8)	48.4 ±9.3	46.4 ±5.9	NA	134.9±22.5			Previously failed adequate conservative treatment	
Schauer 2014 [50]	SG	USA	RCT	49	11 (22)	47.9 ±8	36.2 ±3.9	NA	106.8±14.9	3	1- Fasting glucose	1- Age ranging from 20 to 60years, 2-A glycated hemoglobin level of more than 7.0%, 3- A body-mass index BMI of 27 to 43.	"Among individuals with uncontrolled type 2 diabetes and obesity, a combination of three years of intensive medical therapy with bariatric surgery led to a higher proportion of patients achieving glycemic control compared to those undergoing medical therapy alone. Examination of secondary outcomes, such as body weight, usage of glucose-lowering medications, and quality of life, also indicated favorable outcomes in the surgical groups after three years, in contrast to the group solely receiving medical therapy."
	gastric bypass			48	21 (42)	48.3 ±8.4	37±3.3	NA	100.6±16.5				
Wallenius 2017 [51]	SG	Sweden	RCT	15	8 (5.3)	51.9 ± 1.9	36.9 ± 0.7	55.7 ± 2.1	109. ±3.4	1	Glucose, insulin, and GLP1 levels in surgery time and after a follow-up period of two days, three weeks, and one year.	1-BMI 35–50 kg/m^2^, 2-Age 18–60 years, 3-T2DM requiring any available diabetes medications, but not only dietary regimen.	“LRYGB and LSG show very similar effects on glycemic control, despite lower GLP-1 levels and inferior BMI decrease after LSG.”
	gastric bypass			18	8 (44)	51.2 ± 1.6	38.6 ± 0.8	61.8 ± 3.9	112. ±3.6				

Quality of the Included Studies 

**Figure 2:**
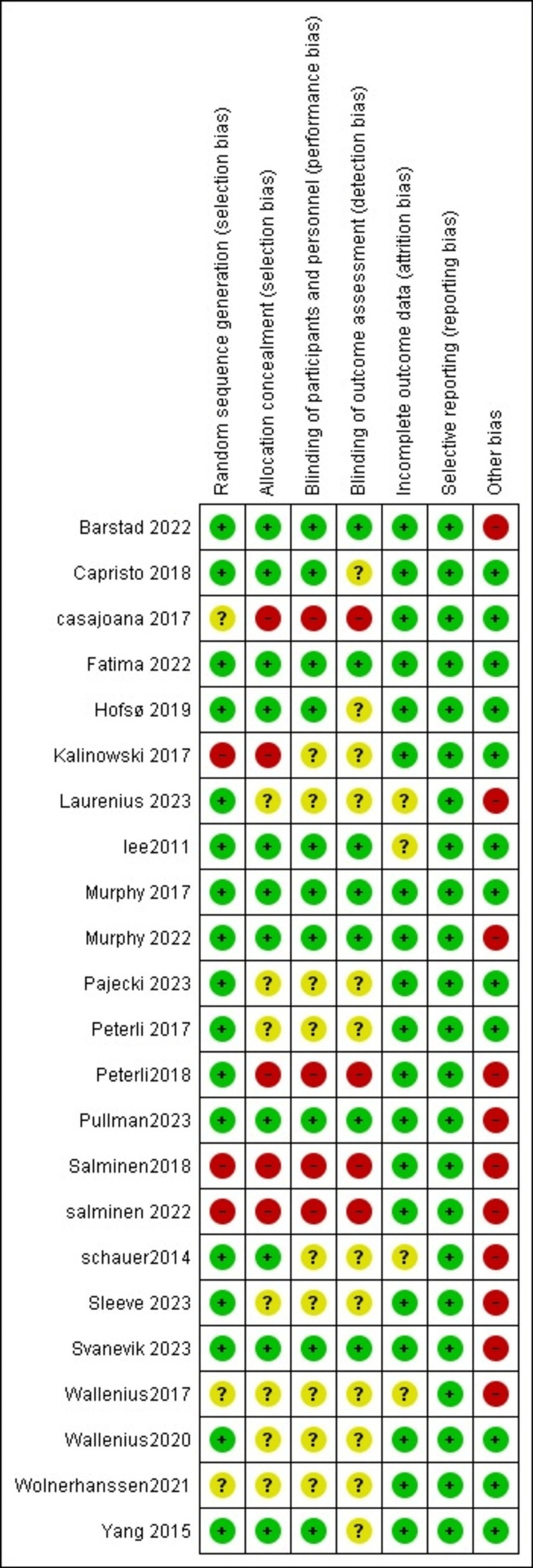
ROB summary [29-51] ROB: Risk of bias

**Figure 3:**
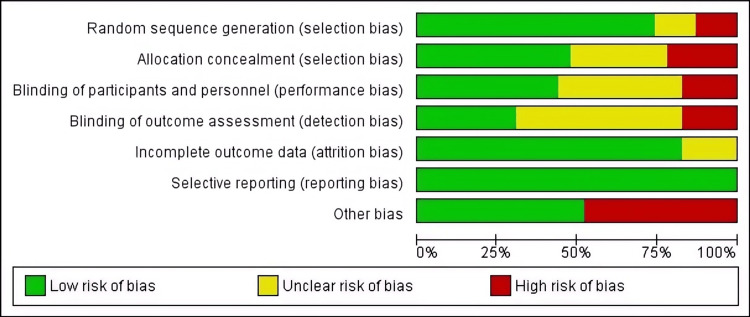
Risk of bias graph

The included RCTs ranged in quality. Two studies were found to have a low ROB in all their characteristics [32,47]; while two others had a low risk in all domains except for the "Other bias" domain [36,45]. Only two studies were found to have a high risk in all domains except for two [40,49]. Overall, most of the studies had a low risk in terms of random sequence. The next three domains, namely allocation concealment, blinding of participants, and outcome assessment, were mostly categorized as either low or unclear risk. A graph showing the ROB is presented in (Figures 2, 3).

 

Efficacy Outcomes

**Figure 4:**
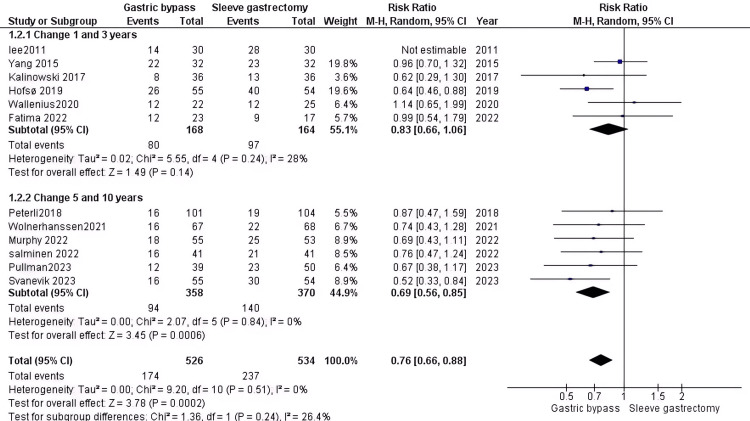
Diabetes remission [32-34,36,38-44,46] The data has been presented in numbers and percentages.

Diabetes remission: Our analysis shows that the group who underwent RYGB surgery had better outcomes compared to the SG group (RR=0.76, 95% CI (0.66 to 0.88), P= 0.0002). The obtained data were homogenous (P=0.51)after the leave-one-out method was applied . For the 1-3 year period, six studies comprising 332 DM patients showed insignificant results (RR = 0.83, 95% CI (0.66 to 1.06), P value = 0.14) and homogeneity (P=0.24, I2 = 28%). For the 5-10 year period, six studies comprising 728 DM patients showed significant results (RR = 0.69, 95% CI (0.56 to 0.85), P value = 0.14) and homogeneity (P=0.84, I2= 0%) after the use of leave one out method (Figure 4).

**Figure 5:**
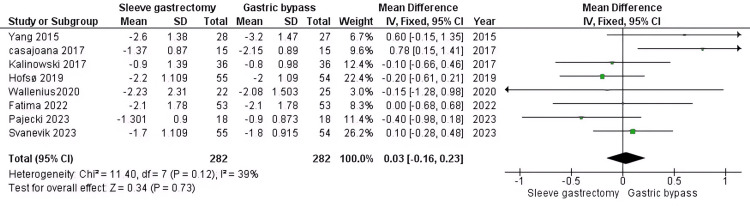
HbA1c [31-34,37,41,42,44] The data has been represented as mean ± SD.

HbA1c: Our study shows that there is no significance between the group who underwent RYGB compared to the other group (MD=0.03, 95% CI (-0.16 to 0.23), P= 0.73). The pooled data were homogenous (P=0.12, I2 = 39%) (Figure 5).

**Figure 6:**
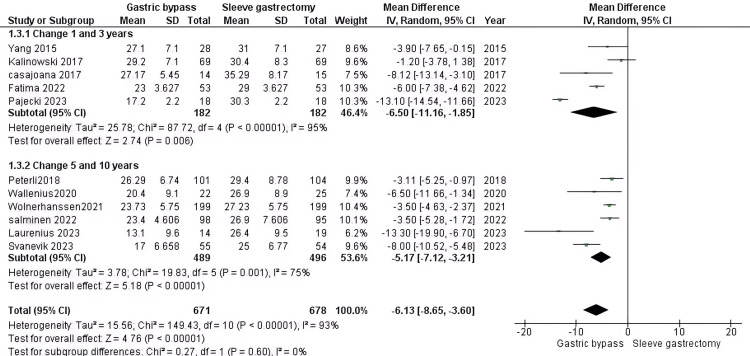
Total body weight loss [31,32,34,35,37,38,40-44] The data has been represented as mean ± SD.

Total body weight loss: Our findings suggest that RYGB resulted in a significantly higher reduction than the other group (MD = -6.13, 95% CI (-8.65 to -3.6), P > 0.00001). However, the pooled data displayed considerable heterogeneity (P > 0.00001, I2 = 93%). During the 1-3 year follow-up period, our analysis incorporated five studies comprising 364 patients with DM. The results were significant differences favoring RYGB (MD = -6.5, 95% CI (-11.16 to -1.85), P > 0.00001) and heterogenous (P > 0.00001, I2 = 95%). During the 5-6 year follow-up period, our analysis incorporated six studies comprising 985 patients with DM. The results were significant differences favoring RYGB (MD = -5.17, 95% CI (-7.12 to -3.21), P > 0.00001) and heterogenous (P = 0.001, I2 = 75%) (Figure 6).

**Figure 7:**
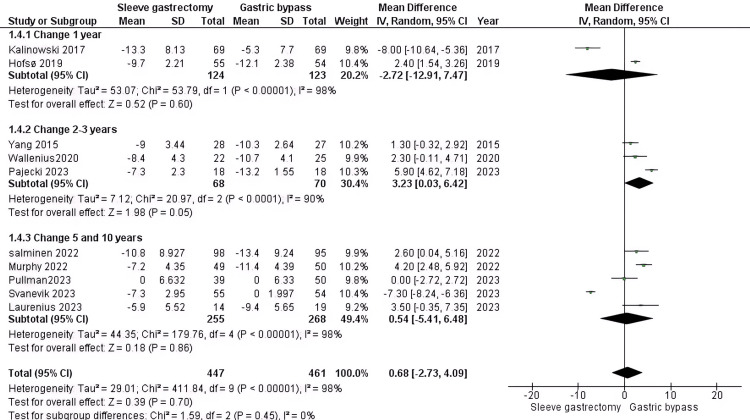
BMI [33-37,39,41,42,44] The data has been represented as mean ± SD.

BMI*:* Our findings indicate that there is no significant difference between the group who underwent RYGB surgery related to the SG group (MD = 0.68, 95% CI (-2.73 to 4.09), P = 0.7). At the 1-year mark, with two studies involving 247 patients with T2DM, the results showed non-significant differences (MD = -2.72, 95% CI (-12.91 to 7.47), P = 0.6), and significant heterogeneity (P > 0.000001, I2 = 98%). In the 2-3 year period, across three studies with 138 DM patients, the results were significant (MD = 3.23, 95% CI (0.03 to 6.42), P = 0.05) with notable heterogeneity (P > 0.00001, I2 = 90%). For the 5-10 year duration, involving five studies with 523 DM patients, the results were non-significant (MD = 0.54, 95% CI (-5.41 to 6.48), P = 0.86) and exhibited substantial heterogeneity (P > 0.00001, I2 = 98%) (Figure 7).

**Figure 8:**
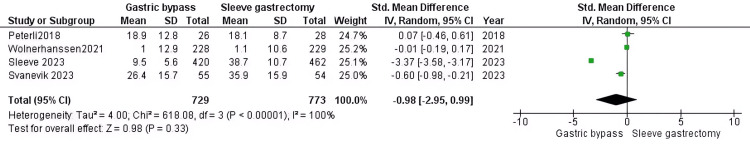
QoL [29,38,41,43] The data has been represented as mean ± SD.

QoL: According to our analysis, there is no statistically significant difference between the group that underwent RYGB and the SG group. The MD was -0.98, with a 95% CI of (-2.95 to 0.99), and a P-value of 0.33, indicating no substantial distinction. The data exhibited homogeneity (P > 0.00001, I2 = 100%) (Figure 8).

Safety Outcomes

**Figure 9:**
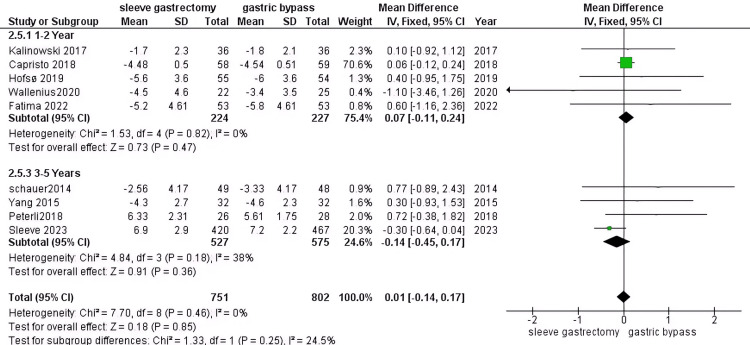
Fasting glucose [30,31,33-35,40,43,45,51] The data has been represented as mean ± SD.

Fasting glucose (mmol): Based on our research, we found no significant difference between the two groups in terms of their outcomes (RR = 0.01, 95% CI (-0.014 to 0.17), P=0.85). However, when the data was pooled, we observed homogenous results (P = 0.46, I2 = 0%). During the 1-2 year study period, we conducted five studies involving 451 DM patients. Our results showed no significant difference among the groups (RR = 0.07, 95% CI (-0.11 to 0.24), P > 0.00001). Furthermore, the data was homogenous (P = 0.82, I2 = 0%). During the 3-5 year study period, we conducted four studies involving 1,102 DM patients. Our analysis revealed a significant difference favoring RYGB (RR = -0.14, 95% CI (-0.45 to 0.17), P = 0.36), and the data was homogenous (P = 0.18, I2 = 38%) (Figure 9).

**Figure 10:**
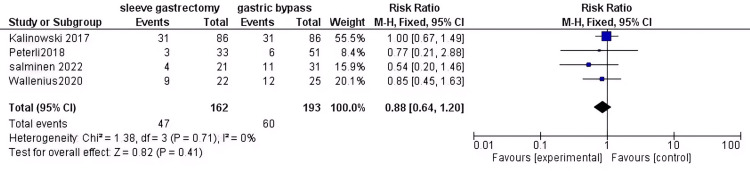
Dyslipidimia [34,38,40,42] The data has been represented as numbers and percentages.

Dyslipidemia: After conducting a thorough analysis of data from 355 patients with DM, we discovered no significant difference in risk between the two groups (RR = 0.88, 95% CI (0.64 to 1.2), P = 0.41). Additionally, the data was found to be homogeneous (P = 0.71, I2 = 0%)(Figure 10).

**Figure 11:**
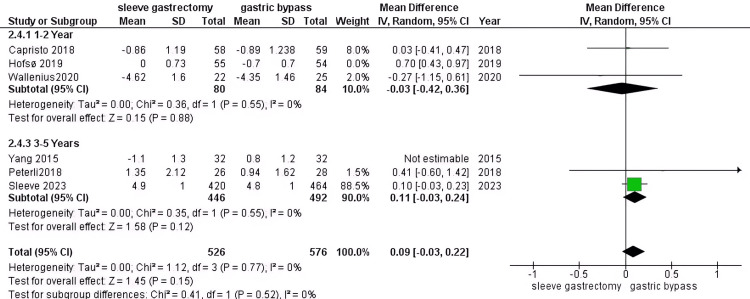
Total cholesterol [29,30,33,38,42,44] The data has been represented as mean ± SD.

Total cholesterol (mmol): Our finding included 1,275 patients with T2DM and obesity, and we found no significance in relation to total cholesterol with mmol between the RYBG and the SG as the RR = -0.13, 95% CI (-0.68 to 0.41), P value=0.63). The result was found heterogeneous and couldn’t be resolved by any test (P < 0.00001) (Figure 11).

**Figure 12:**
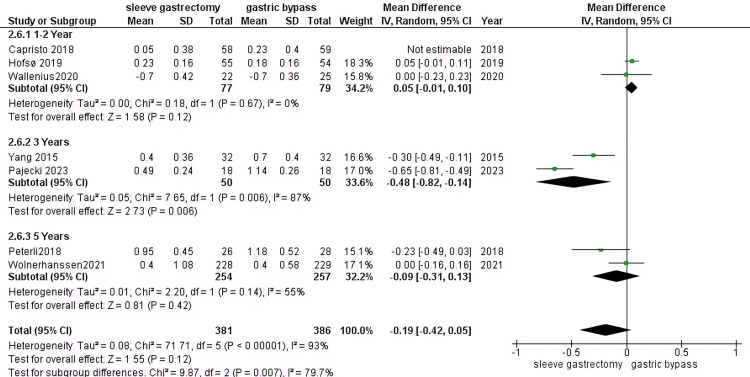
HDL [30,33,37,38,42-44] The data has been represented as mean ± SD. HDL: High-density lipoprotein

HDL (mmol): Our analysis reveals no significant difference between patients undergoing RYGB surgery and those in the SG group (MD = -0.18, 95% CI (-0.38 to 0.01), P = 0.06). The pooled data exhibited considerable heterogeneity (P > 0.00001, I2 = 92%). Subgroup analyses for 1-2 years (three studies, 272 DM patients) yielded non-significant results (MD = -0.04, 95% CI (-0.2 to 0.12), P = 0.6) and (P = 0.01, I2 = 77%). At 3 years (two studies, 100 DM patients), the results were significant (MD = -0.48, 95% CI (-0.82 to -0.14), P = 0.05) and (P = 0.006, I2 = 87%). For the 5-year duration (two studies, 511 DM patients), results were non-significant (MD = 0.009, 95% CI (-0.31 to 0.13), P = 0.42) with heterogeneity (P = 0.14, I2 = 55%) after leave-one-out method (Figure 12).

**Figure 13:**
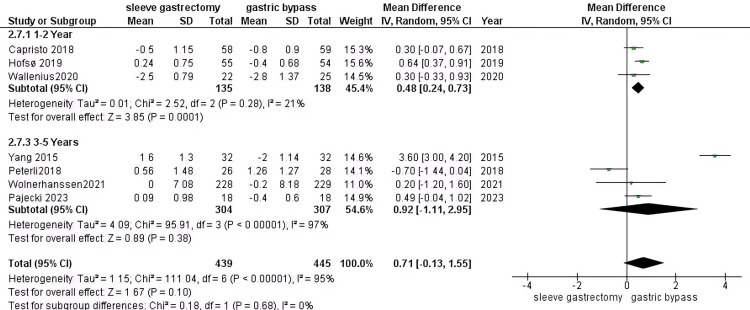
LDL [30,33,37,38,42-44] The data has been represented as mean ± SD. LDL: Low-density lipoprotein

LDL (mmol): Our findings indicate that there is no significant difference between the group who underwent RYGB surgery compared to the SG group (MD = 0.71, 95% CI (-0.13 to 1.55), P = 0.1). During the 1-2 year follow-up period, our analysis incorporated three studies comprising 272 patients with DM. The results were significant differences (MD = 0.48, 95% CI (0.24 to 0.73), P value = 0.0001) and homogenous data (P = 0.28, I2 = 21%). During the 3-5 year follow-up period, our analysis incorporated four studies comprising 611 patients with DM. Non-significant results were found (MD = 0.92, 95% CI (-1.11 to 2.95), P value = 0.38) and heterogeneous data (P > 0.00001, I2 = 97%) (Figure 13).

**Figure 14:**
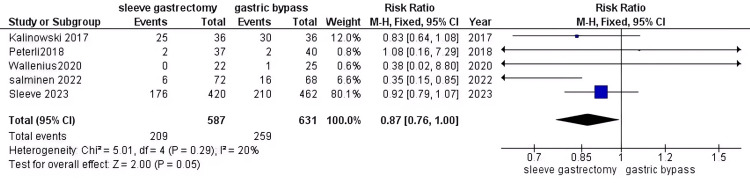
HTN [29,34,38,40,42] The data has been represented as numbers and percentages. HTN: Hypertension

HTN: After conducting a thorough analysis of data from 468 patients with DM, we discovered no significance in risk between the two comparator groups (RR = 0.87, 95% CI (0.76 to 1), P = 0.05). Additionally, the data was found to be homogeneous (P = 0.29, I2 = 20%)(Figure 14).

**Figure 15:**
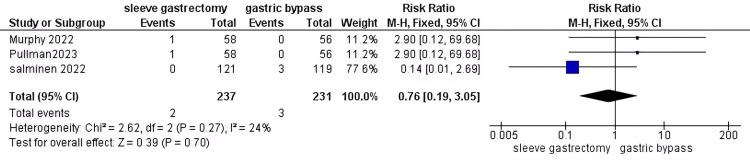
Vomiting [36,39,40] The data has been represented as numbers and percentages.

Vomiting: After conducting a thorough analysis of data from 468 patients with DM, we discovered no significant difference in risk between the two groups (RR = 0.76, 95% CI (0.19 to 3.05), P = 0.7). Additionally, the data was found to be homogeneous (P = 0.27, I2 = 24%)(Figure 15).

Discussion

Our meta-analysis, comprising 23 RCTs, systematically examined the outcomes of RYGB and SG across various follow-up periods: one to three years, three to five years, and over five years. No significance in BMI was observed between the two interventions overall and in individual follow-up periods, except for a marginal significance (p = 0.05) favoring RYGB in the two to three-year interval. For diabetes remission, a pivotal outcome, RYGB demonstrated significant efficacy in the long-term follow-up, ranging from five to ten years, with consistent and homogenous results, positioning RYGB as superior for patients with T2DM. Total body weight loss favored RYGB significantly in all follow-up periods, emphasizing its efficacy for patients requiring substantial weight loss. Efficacy outcomes related to HbA1C and QoL revealed no significance between RYGB and SG. Regarding safety outcomes, HDL exhibited significance only in the three-year period, while HTN occurrence, though non-significant (p = 0.05), warranted further investigation due to limited data in five studies. LDL showed no overall difference, with significance observed only in the one to two-year interval. No other efficacy outcomes, including vomiting and fasting blood glucose, demonstrated statistical significance. In summary, our comprehensive analysis underscores the superiority of RYGB in achieving diabetes remission and total body weight loss over various follow-up periods, with limited differences in other efficacy and safety outcomes while showing superiority for SG at pretension outcomes. Our contribution is deemed significant in advancing the current understanding of T2DM post-bariatric surgery, as we have employed consistent definitions. This approach enhances the comparability of studies, allowing for the interpretation of findings across diverse populations with varying inclusion criteria and from different countries.

The most challenging part in estimating the results of RYGB and SG procedures is that these two procedures are long-term surgeries, and maintaining the connection with the patients and following them up for a long period might be difficult and would result in high costs for the RCT, so we can find that most papers would provide long-term safety results. Despite this fact, many RCTs were published concerning RYGB or SG with limited numbers and short follow-up periods, some focused on diabetic patients alone, and others focussed on all patients who are obese. Higher BMI increases the risk of death and complications for diabetic patients; from our findings, both techniques could efficiently reduce the BMI although no significant difference was found between RYGB and SG [52,53]. Bariatric surgeons are consistently striving to innovate and devise novel procedures rooted in SG principles. The objective is to streamline surgical techniques, mitigating both the surgical and metabolic risks associated with the procedure, all the while preserving favorable outcomes. About the safety of the procedures, it is notable that no fatalities were found to be linked with either the SG or the RYGB which would add comfort to the patients intending to go through these primary bariatric procedures [54].

Our study has several strengths as we are the updated version of the previous meta-analysis that was previously performed as we have a bigger population and more included RCTs. Additionally, we only included RCTs although most of the studies made about both surgeries were observational we wanted to maintain RCTs as our only source of evidence as the RCTs are the gold standard for evidence. We made three different subgroups to ensure the results are followed up in the short and the long term. Many RCTs were excluded due to the very poor study design and the high risk of bias. We also had several limitations, The high heterogeneity of the data from the included studies may lead to misleading results, and the included patient numbers in each of the included RCTs were not similar, in addition to the inadequacy of trials and patients, which should be taken into consideration while interpreting these data. The majority of our studies were single centers with a low number of participants. We can indicate that future research to focus on performing multicenter studies with long follow-up periods to estimate the long-term outcomes with the lowest heterogeneity among patients by investigating the side effects of both of the techniques.

## Conclusions

RYGB demonstrated a notably significant enhancement in diabetes remission for patients with T2DM, particularly in the long-term follow-up, compared to the SG procedure. RYGB also exhibited superior total body weight loss in both short-term and long-term assessments. However, no significant variations were found in other efficacy outcomes between the two procedures. Safety outcomes warrant further investigation due to the lack of data, with HTN being the only parameter with a p-value of 0.05 that is barely significantly lower in SG compared to RYGB. Notably, no significant distinctions were found in HDL, hyperlipidemia, fasting blood glucose, vomiting, LDL, and total cholesterol. Additional research is needed to assess safety outcomes for both surgical approaches comprehensively. The summary of our findings shows the superiority of the gastric bypass without additional side effects which could be used as a guideline for the bariatric surgeons.
